# Comparative Study on Preparation Methods for Transparent Conductive Films Based on Silver Nanowires

**DOI:** 10.3390/molecules27248907

**Published:** 2022-12-14

**Authors:** Jizhe Zhang, Xingzhong Zhu, Juan Xu, Ruixing Xu, Hao Yang, Caixia Kan

**Affiliations:** 1 College of Physics, Nanjing University of Aeronautics and Astronautics, Nanjing 211106, China; 2 Key Laboratory of Aerospace Information Materials and Physics (NUAA), MIIT, Nanjing 211106, China

**Keywords:** Ag nanowires, transparent conductive films, preparation methods

## Abstract

Silver nanowires, which have high optoelectronic properties, have the potential to supersede indium tin oxide in the field of electrocatalysis, stretchable electronic, and solar cells. Herein, four mainstream experimental methods, including Mayer–rod coating, spin coating, spray coating, and vacuum filtration methods, are employed to fabricate transparent conductive films based on the same silver nanowires to clarify the significance of preparation methods on the performance of the films. The surface morphology, conductive property, uniformity, and flexible stability of these four Ag NW-based films, are analyzed and compared to explore the advantages of these methods. The transparent conductive films produced by the vacuum filtration method have the most outstanding performance in terms of surface roughness and uniformity, benefitting from the stronger welding of NW-NW junctions after the press procedure. However, limited by the size of the membrane and the vacuum degree of the equipment, the small-size Ag films used in precious devices are appropriate to obtain through this method. Similarly, the spin coating method is suited to prepare Ag NWs films with small sizes, which shows excellent stability after the bending test. In comparison, much larger-size films could be obtained through Mayer-rod coating and spray coating methods. The pull-down speed and force among the Mayer-rod coating process, as well as the spray distance and traveling speed among the spray coating process, are essential to the uniformity of Ag NW films. After being treated with NaBH_4_ and polymethyl methacrylate (PMMA), the obtained Ag NW/PMMA films show great potential in the field of film defogging due to the Joule heating effect. Taken together, based on the advantages of each preparation method, the Ag NW-based films with desired size and performances are easier to prepare, meeting the requirements of different application fields.

## 1. Introduction

With the flourishing development of wearable and foldable electronics, traditional materials such as indium tin oxide (ITO) cannot satisfy the requirement of potential applications for flexible electronics due to its scarcity of indium and brittleness [1,2,3,4,5,6,7,8]. Metal nanowires, such as gold nanowires, silver nanowires, and copper nanowires fabricated via various methods such as seed-medicated growth method and wet-chemical method, attracted more attention based on the high aspect ratio [9,10]. In recent years, more and more experiments demonstrated that Ag nanowires (NWs) networks could be widely used in wearable electronic devices, touch screens, and solar cells due to their high optoelectronic and physicochemical properties compared with other photoelectric materials [11,12]. Synthesis of Ag NWs with a high aspect ratio has become one of the most favorable approaches to improve the conductivity and transparency of Ag NW-based films. However, the morphology and the distribution of the Ag NWs network, as well as the combination of Ag NWs and substrate, are also essential to the performance of the obtained films. Relative to the preparation of Ag NWs, how to integrate Ag NWs into the flexible matrix is particularly important and needs intensive study [13,14,15,16,17,18,19,20,21,22,23].

To solve the problem of practical application, coating technology was widely applied in the manufacture of flexible transparent conductive films (FTCFs), which include drop casting, screen printing techniques, inkjet printing techniques, template-assisted assembly, etc. [24,25,26,27,28,29,30,31,32,33]. However, these methods, especially drop casting and printing techniques, are too complex to fabricate inconsistently conducting films [34,35,36,37]. In comparison, four methods with the conventional procedure, which include Mayer-rod coating, spin coating, spray coating, and vacuum filtration attracted a lot of attention. The Mayer-rod coating method was a process that Ag NWs solution was poured onto the substrate and scraped flat by Mayer rod (a bar enwound by metal wires) [38]. Yang Li et al. fabricated Ag NWs/PDAC/GO film within resistance of 10 Ω and transparency of 91% by this method [39]. Spin coating was a process to form a uniform film on a revolving platform via centrifugal force [40]. Ye Zhang et al. fabricated Ag NWs film with high conductivity and low light extinction in the visible area by spin coating [41]. For the spray coating method, the Ag NW dispersion was atomized via the nozzle under the action of compressed gas. Ag NWs film had no more need for high-temperature annealing after the spray coating process [42]. Sae Mi Lee et al. fabricated Ag NWs/GO/OCL films which have resistance of 15 Ω, transparency of 90%, and excellent stability via spray coating [43]. In the vacuum filtration process, Ag NWs dispersion was passed through the filtration funnel and remained on the membrane to form the Ag NWs network. The films could be obtained after the transfer of the Ag NWs network from membrane to the substrate by tablet press. This process can fabricate uniform conductivity film with sample procedures. Xu et al. prepared a transparent conductive film based on an Ag NWs network under a vacuum filtration process [44]. The transparency of this film can be adjusted from 65.6 to 87.5% and sheet resistance from 177.3 to 4.95 Ω/sq by controlling the density of the Ag NWs solution. In general, Ag NW-based films with excellent conductivity and transparency could be obtained through these four methods. However, based on the difference between the preparation process of each method, the obtained Ag NW-based films possess a lot of discrepancies in size, morphology, uniformity, and stability performance, which are lacking systemically in studies in the past. Clarifying the advantages and disadvantages, as well as the corresponding application fields of each method are urgently needed to promote the application of Ag NW-based films.

In this work, the same purified Ag NWs were employed as the basic materials to produce transparent conductive films. Ag NW-based FTCFs prepared through different methods, including Mayer-rod coating, spin coating, spray coating, and vacuum filtration methods, are compared with each other from optoelectronic properties and morphology to explore their parallel environment of applications. After characterizing the properties of these obtained FTCFs, more details in surface morphology, and sheet resistance distribution through four methods was analyzed to clarify the specialty of each method. For further explore application in other fields, FTCFs were soaked by NaBH_4_ and coated by polymethyl methacrylate (PMMA), showing excellent performance in bending tests and film-heating experiments.

## 2. Results and Discussion

**Figure 1:**
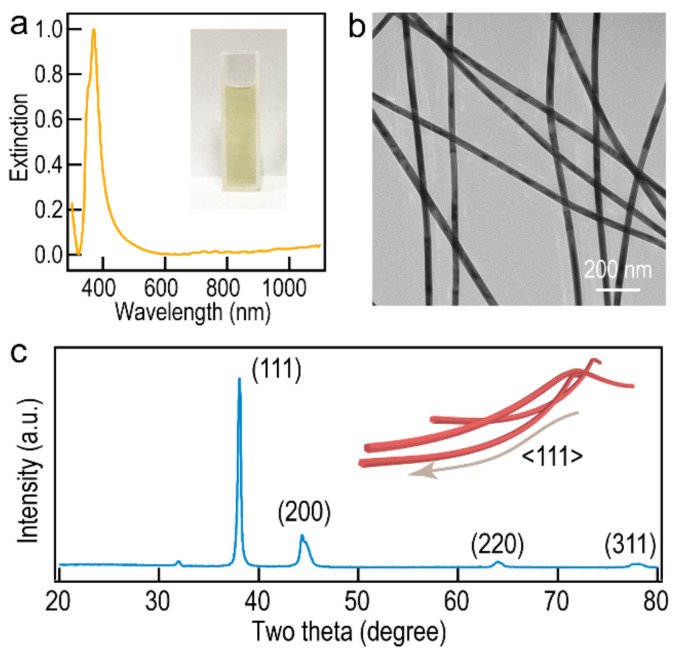
(**a**) Extinction spectrum of the purified Ag NWs. The inset is the photograph of the purified Ag NWs. (**b**) TEM image of the purified Ag NWs. (**c**) XRD pattern of purified Ag NWs. The inset is the model of Ag NWs.

On the basis of our previous work, Ag NWs are synthesized by a modified polyol method and further purified through selective precipitation [45]. As shown in Figure 1a, the UV spectrum exhibits a special peak of Ag NWs at 370 nm, which is attributed to the transverse longitudinal surface plasmon mode of one-dimensional silver nanostructures. In addition, the plasmon effect of the Ag NWs leads to the high transparency of Ag NW-based films in visible and near-infrared regions [46]. TEM characterization shows that the Ag NWs have a uniform morphology and a narrow dispersion in sizes, with an average diameter of 32 nm and a length of up to 35 µm (Figure 1b and Figure S1). The high aspect ratio (>1000) ensures the formation of the Ag NW network, which endows the Ag NW-based films with high conductivity for electronics applications. The XRD pattern suggests that the Ag NWs have a face-centered cubic structure (Figure 1c). The two theta peaks at 38.1°, 44.6°, 64.1°, and 77.9° corresponded to (111), (200), (220), and (311) reflection lines of the silver nanostructure, respectively [47]. Furthermore, there are no characteristic peaks of impurities (AgO), indicating the purity of Ag NWs.

**Figure 2:**
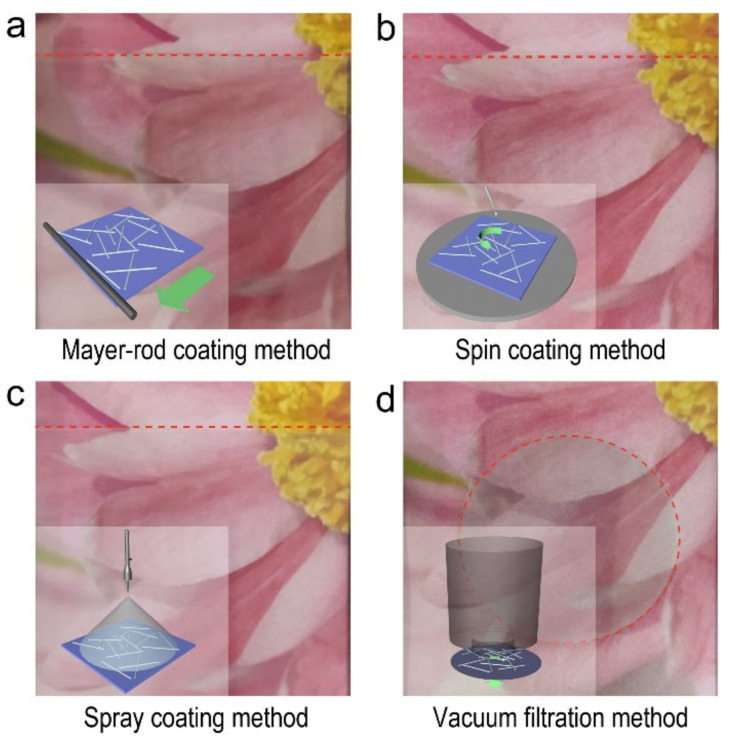
Photographs of Ag NW-based films prepared through (**a**) Mayer-rod coating, (**b**) spin coating, (**c**) spray coating, and (**d**) vacuum filtration methods. The inset is the corresponding diagrams of these four methods.

The Ag NW-based FTCFs could be fabricated via Mayer-rod coating, spin coating, spray coating, and vacuum filtration methods, respectively. Figure 2a–d shows the schematic diagrams and the corresponding photographs of the prepared Ag NW-based FTCFs. Compared with industrialized printing technology, these approaches did not rely on an ambitious experimental environment and equipment. The digital photographs show that these four Ag NW-based FTCFs are highly transparent with nearly colorless. Interestingly, it is clear that the entire PET substrate is covered by Ag NWs using Mayer-rod coating, spin coating, and spray coating methods. However, the circular film is obtained through the vacuum filtration method, which is limited by the configuration of the filter. In addition, the detailed digital photographs of Ag NW-based FTCFs fabricated by Mayer-rod coating and spray coating methods reveal that the Ag NWs are unevenly distributed on PET substrate with obvious accumulation, which could be ascribed to the thickness of the films and the evaporation rate of the solvent during the subsequent drying process (Figure S2) [23,24,25,26,27,28,29,30,31,32,33,34,35,36,37,38,39,40,41,42,43,44].

**Figure 3:**
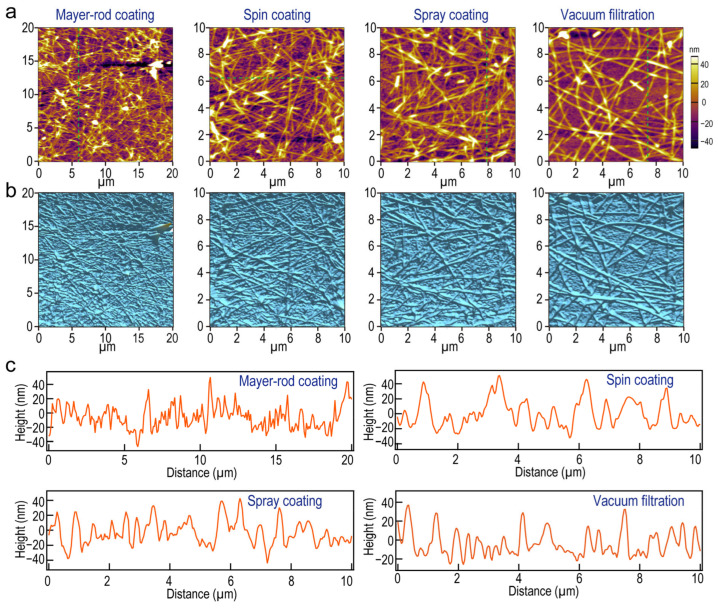
(**a**) AFM topographical and (**b**) three-dimensional images of Ag NW-based films prepared through vacuum filtration, Mayer-rod, spin coating, and spray coating methods, respectively. (**c**) The height profile of the Ag NWs indicated with the green dashed line in (**a**).

The surface topography and roughness of the four Ag NW-based films were investigated using SEM characterization and atomic force microscopy (AFM) operated in the tapping mode. It is clear that the Au NWs were distributed uniformly on the surface of the PET substrate in large areas (Figures S3 and S4). The high roughness would lead to interlayer shorting, high leakage current, and low quantum efficiency in electronic devices, which hinders Ag NW-based films from being compatible with efficient devices [48]. Lower roughness allows Ag NW-based films to satisfy the requirement of flexible devices. Figure 3a,b shows the AFM topographical image of the Ag NW-based films prepared by these four methods in different views. The overlap of Ag NWs and the undulation of the film surface could be clearly observed. The height line scan data reveals the uniform distribution of the height of Ag films along the green dashed line (Figure 3c). Additionally, the root mean square (RMS) is one of the factors to reflect the comprehensive roughness of the films directly, which is the root mean square along the sampling length. The RMS value of the Ag NWs film produced through the vacuum filtration method is 18.2, smaller than those of films prepared by the Mayer-rod coating method (25.4), spin coating method (20.8), and spray coating method (18.22), indicating the enhanced surface smoothness of the vacuum filtration method. The NWs-NWs junctions in physical entanglement are penetrated with external pressure, enhancing the adhesion and integrity between the Ag NWs and the substrate effectively. The higher RMS value of the Ag NW-based films prepared by the Mayer-rod coating method is possibly attributed to the squeeze of Ag NWs by the Mayer-rod during the scraping process. For instance, I G. Serrano used the nanoimprint technique [49] and lateral spin devices [50] to fabricate graphene-based flexible electronics with ultra-smooth surfaces, in which RMS decreases below 10 nm. The present results indicate that the as-prepared Ag NW-based films with smooth and flat surfaces can meet the requirement of further practical application in touch screens or organic light-emitting diodes [51].

**Figure 4:**
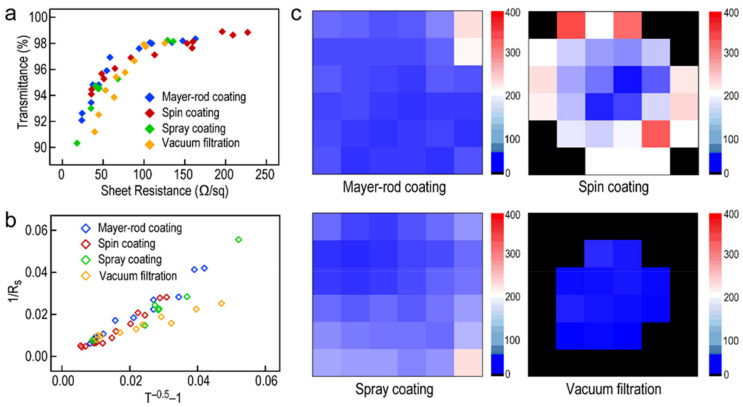
(**a**) Plots of optical transmittance (*λ* = 550 nm) versus sheet resistance for Ag NW-based films by four methods. (**b**) Plots of 1/*R_s_* as a function of (*T(λ)*^−0.5^ − 1) for these four films. (**c**) Sheet resistance maps of Ag NW-based films fabricated by Mayer-rod method (6 × 6 cm^2^), spin coating method (6 × 6 cm^2^), spray coating method (6 × 6 cm^2^), and vacuum filtration method (4 × 4 cm^2^), respectively.

In general, sheet resistance and transparency are two key factors to estimate the optoelectronic properties of Ag NW-based films. Sheet resistance is an essential index relative to electronic conductivity, permittivity, and electromagnetic shielding efficiency for observing optoelectronic properties. Transparency also can reflect the optoelectronic property in the visible region to develop applications. The obtained films, especially spray coating and spin coating method fabricated ones, showed excellent transparency in the visible region (Figure S5). Figure 4a exhibits plots of transmittance versus sheet resistance of the Ag NW-based FTCFs fabricated through these four methods. These films show outstanding conductivity (from 10 to 200 Ω sq^−1^) and transmittance (from 90 to 98%). Only slight differences in these Ag NW-based films could be observed. In general, the transmittance and sheet resistance of a thin metallic film may be expressed as [51,52]:

(1) $$T\left(\lambda \right)=\;\left(1+\;\frac{Z_{0}}{2R_{S}}\;\frac{\sigma_{OP}\left(\lambda \right)}{\sigma_{DC}}\right)$$

where *λ* is the wavelength, *T*(*λ*) is transparency at *λ* nm, and *Z*_0_ is the wave impendence of free space (377 Ω). *σ_OP_*(*λ*) is the optical conductivity at *λ* nm. *σ_DC_* is the direct current conductivity of the film. The FOM is expressed as the ratio of the direct current conductivity to the optical conductivity (*σ_DC_*/*σ_OP_*(*λ*)), which has been used in polymer, carbon nanotubes, and Ag NW meshes. To further evaluate the optoelectrical performance of these Ag NW-based FTCFs, we calculated the *FOM* according to the variant form of Equation (1) as follows:


(2) $$\frac{1}{R_{S}}=\;FOM\;\frac{T\;{\left(\lambda \right)}^{-0.5}\;-\;1}{188.5}$$

*FOM* about all films can be obtained via slope linear fitting the plot of 1/*R_s_* versus (*T*(*λ*)^−0.5^ − 1). The conductivity of the films is increased as the value of *FOM* increased. As shown in Figure 4b, the values of *FOM*, which include the Mayer-rod coating, spin coating, spray coating, and vacuum filtration, are 69.7, 81, 82.9, and 112.2, respectively. From the view of *FOM*, the Ag NW-based film fabricated by the Mayer-rod coating method is relatively poor in conductivity performance, which accords with the surface morphology of the Ag NW layers. Therefore, the conductivity of the obtained Ag NW-based films is highly associated with the employed preparation method.

In addition, the optoelectrical properties of the Ag NW-based films are directly determined by the distribution of Ag NWs. To be more suitable for practical application, the uniformity of the Ag NWs layer is another essential parameter to determine the applicability of the Ag NW-based FTCFs. Figure 4c shows the area maps of sheet resistance for the Ag NW-based FTCFs synthesized by these four methods, respectively. The sheet resistance was measured at the same areas after fabricating Ag NW layers through different methods on the same PET substrates (Figure S6). It is clearly revealed that there is an obvious difference in the distribution of sheet resistance among the obtained FTCFs. The Ag NW-based FTCFs produced through Mayer-rod coating and spray coating methods possess uniform sheet resistance distribution on all regions of the films. Controlling the pull-down speed and force in the Mayer-rod coating process, as well as the spray distance and travelling speed among the spray coating process is the key to fabricating uniform Ag NW films. In comparison, only the center proportion of the film produced by the spin coating method show uniform sheet-resistant distribution owing to the effect of centrifugal force. The spin coating method cannot meet the requirement of large-size film due to the deformation of the flexible substrate during the rapid rotation. Furthermore, the Ag NW-based films obtained by the vacuum filtration method showed the highest uniformity. However, large-size films are difficult to synthesize under the limitation of the size of the membrane and the vacuum degree of equipment [11]. In conclusion, the Mayer-rod coating and spray coating method meet the great prospect of realizing the fabrication of large-scale films (Figure S8). In contrast, the spin-coating and vacuum filtration methods are suitable for the application of small-scale precision devices.

**Figure 5:**
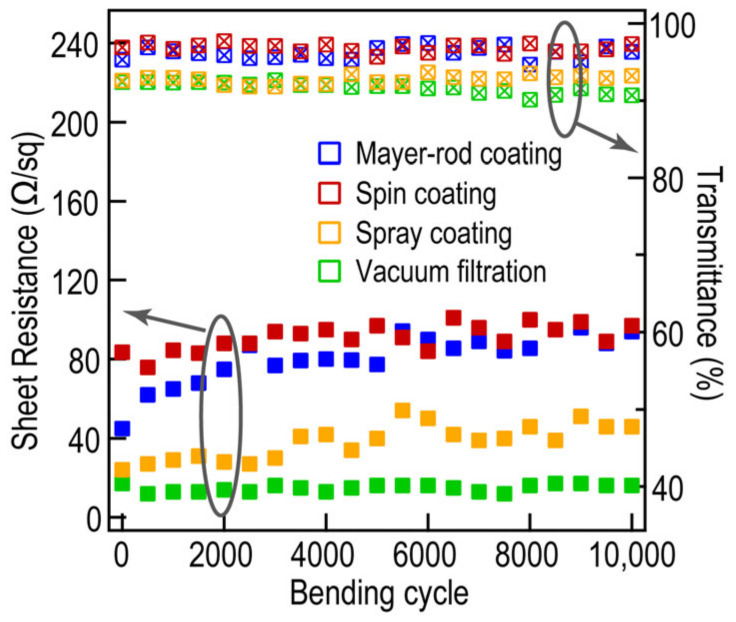
Sheet resistance and optical transmittance for the Ag NW/PMMA films by different methods after 10,000 bending cycles under 50% strain.

To further improve the optoelectronic property and stability of the Ag films, the NaBH_4_ and PMMA layer are employed to enlarge the contact area between Ag NWs and to prevent the Ag NWs from oxidation. As shown in Figure S7, the FOM factors of four Ag NW/PMMA films are effectively increased to 90, 107, 224, and 137, respectively. With the development of flexible devices, Ag NWs as an important component of flexible electronic devices have shown the potential to replace or even surpass traditional materials [43]. Furthermore, an additional bending-release cycling test is conducted to explore the mechanical stability of these four Ag NW/PMMA films (Figure 5). The sheet resistance and transmittance of the Ag NW/PMMA films obtained through the spin coating and vacuum filtration methods remain stable with a little fluctuation after 10 000 bending-release cycles. However, those of Ag NW/PMMA films produced by Mayer-rod and spray coating methods have an obvious increase. The difference in the stability for the Ag NW/PMMA films could be attributed to the much stronger welding of NW-NW junctions from basic Ag NWs network in the vacuum filtration method [53,54]. Taken together, the diverse experimental procedure leads to a slight discrepancy in performance, but macroscopically, the films prepared by all these approaches have excellent physical flexibility. From this point of view, four experimental methods will not affect the physical flexibility of Ag NWs during the experimental process.

**Figure 6:**
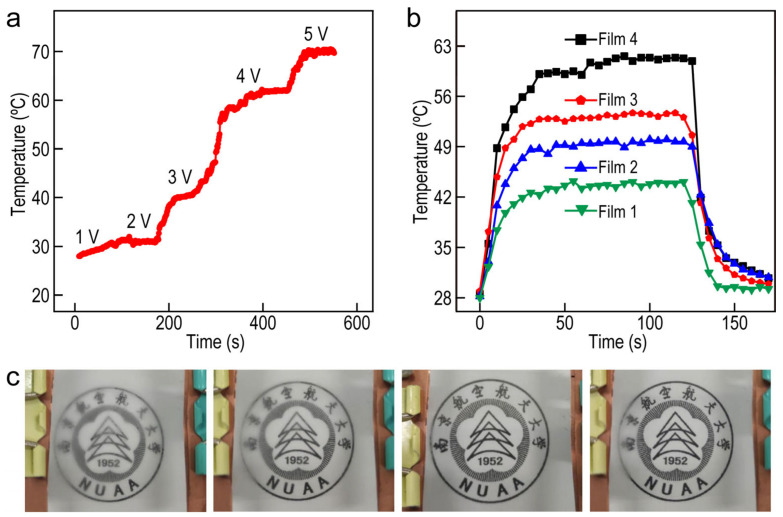
(**a**) The temperature evolution of the Ag NW/PMMA FTCFs with stepwise voltage increases from 1 to 5 V. (**b**) Temperature increase profiles of the Ag NW/PMMA FTCFs with different Ag NW concentrations. (**c**) Photographs of the defogging process at a voltage of 3 V using Ag NW/PMMA FTCFs at different time points.

On the basis of the conductivity and stability of Ag NW/PMMA films, the Joule heating and defogging properties of the oriented Ag NW-based electrode were also studied. We selected the films prepared by spray coating for the following characterization experiment. Figure 6a presents the time-dependent temperatures at various applied voltage, while the voltage was increased by 1 V every 100 s until the heater failed at 6 V bias voltage. As the voltage increased from 1 to 5 V, the temperature of the films increased rapidly and reached a steady-state temperature within 50 s. The steady-state temperature of the film could reach 72.5 °C. The temperature–time curves of the films with different concentrations of Ag NWs under the applied voltage of 3 V are exhibited in Figure 6b. The temperature of these films increases rapidly and reached a steady-state temperature within 30 s. Additionally, the temperature stabilizes within 100 s and drops to the initial state within 20 s while the applied voltage is cutting off. The corresponding relationship between voltage and temperature promotes its application value in defogging, which can be intuitively reflected in Figure 6c. The characterization of defogging performance and physical stability of films elucidates that the films prepared by these approaches have the potential to be applied in complex environments. Meanwhile, varied synthesis approaches of the Ag NW-based FTCFs provide more opportunities to explore the potential applications in the field of electromagnetic interference shielding, touch screens, solar cells, and windshield-glass heaters.

## 3. Materials and Methods

### 3.1. Chemicals

Sodium chloride (NaCl, 99%), sodium bromide (NaBr, 99%), and polyvinylpyrrolidone (PVP, Mw = 1,300,000) were purchased from Sigma–Aldrich (St. Louis, MO, USA). Ethanol (99%), acetone (99%), isopropanol (99%), ethylene glycol (EG, 99%), and sodium borohydride (NaBH_4_, 99%) were purchased from Sinopharm Chemical Reagent (Shanghai, China). Polymethyl methacrylate (PMMA) (AR-P 672.08) was obtained from Allresist Co., Ltd. (Strausberg, Germany) AgNO_3_ (99%) was purchased from Alfa Aesar (Waltham, MA, USA). Deionized water with a resistivity of 18.2 MΩ cm was gained from the Direct–Q5 ultraviolet water purification system.

### 3.2. Preparation and Purification of the Ag NWs

Ag NWs were prepared by the modified polyol method. First, PVP (600 mg) was added to the EG (20 mL) in the oil bath at 160 °C with magnetic stirring. After 5 min, 120 µL of 300 mM NaCl solution and 60 µL of 300 mM NaBr solution were added into the flask. Subsequently, 10 mL of 25 mg/mL AgNO_3_ solution was added dropwise via a syringe pump at a rate of 200 µL/min. After the addition was completed, the resultant Ag NWs were cooled to room temperature with water flushing immediately. Afterward, the acetone was slowly dispersed in the resultant solution, and precipitation of Ag NWs was observed. The supernatant was dislodged from precipitation of Ag NWs after allowing the solution to stand for 10 min. The aggregated Ag NWs were redispersed in 50 mL water containing 0.5 wt% PVP. This procedure was repeated five times to promise highly pure Ag NWs. The product was diluted with 30 mL water for further use.

### 3.3. Fabrication of Ag NW Films by Mayer–Rod Coating Method

PET with a size of 6 × 6 cm^2^ and a thickness of 150 µm was used as the substrate for the fabrication of FTCFs. The protective layer on the PET was peeled off, followed by the treatment of a plasma cleaning machine. The purified Ag NW solution was centrifuged at 2300× *g* rpm for 10 min and the precipitate was dispersed into the mixture of isopropanol and water in a volume ratio of 4:1. Afterwards, the as-prepared Ag NW solution was dripped onto the PET substrate slide. A Mayer-rod (50 µm) was then quickly pulled down the solution, spreading it across the substrate into a thin, uniform film, followed by being dried in a vacuum oven at 60 °C for 10 min.

### 3.4. Fabrication of Ag NW Films by Spin Coating Method

PET with a size of 6 × 6 cm^2^ and a thickness of 150 µm was used as the substrate for the fabrication of FTCFs. After treatment of the substrate and centrifugation of Ag NWs, Ag NWs were spin-coated on the PET substrate at 550 rpm for 18 s and dried in a vacuum oven at 60 °C for 10 min.

### 3.5. Fabrication of Ag NW Films by Spray Coating Method

PET with a size of 6 × 6 cm^2^ and a thickness of 150 µm was used as the substrate for the fabrication of FTCFs. Typically, a spray gun with an aperture of 0.2 mm was used and the sprinkling area was adjusted to fit the substrate. After treatment of the substrate and centrifugation of Ag NWs, Ag NW solution was sprayed on the substrate under compressed air pressure of 30 psi at a distance of 15 cm, followed by natural drying.

### 3.6. Fabrication of Ag NW Films by Vacuum Filtration Method

The Ag NW solutions with different concentrations were dispersed in deionized water. The thin film was constructed by filtering down the nanowires from the dispersion onto a cellulose porous membrane (pore size 220 nm) via vacuum filtration for 2 min. At last, a PET substrate (4 × 4 cm^2^) was placed on the side of the filter membrane with captured Ag nanowires and then pressed against the back side of the filter membrane using a tablet press with 10 Mpa pressure for 1 min. On this basis, the Ag NW-based FTCF was completed through the vacuum filtration method.

### 3.7. Fabrication of Ag NW/PMMA Films

The prepared Ag NW-based FTCFs were dipped in 0.5 M NaBH_4_ (ethanol:H_2_O = 1:1) for 1 min to improve the NW-NW junctions, followed by washing with ethanol/deionized water and drying with argon gas. Afterward, the PMMA protection layer was produced by spin-coating the PMMA (15 wt% in acetone) solution at 500 rpm and 3600 rpm for 30 s on the film surface, respectively. The Ag NW/PMMA film was obtained after drying in a fume hood at room temperature for 10 min.

### 3.8. Instrument and Characterization

The extinction spectra were obtained on the Shimadzu UV–3600 plus ultraviolet/visible/near-infrared spectrophotometer (Shimadzu, Kyoto, Japan). Transmission electron microscopy (TEM) imaging was carried out on an FEI Tecnai 12 microscope operated at 120 kV. The sheet resistance was measured on the four–probe methods (RTS–8) based on the linear four–probe technology. PET was treated by the plasma cleaner (CPC–A). Atomic force microscopy (AFM) images were got by Asylum Research MFP–3D–SA with repulsive force in tapping mode. X-ray diffraction (XRD) image was obtained via a Panalytical Empyrean diffractometer using Cu Kα radiation (*λ* = 1.5406 Å) in the ranges of 20° to 80°.

## 4. Conclusions

In this work, the performance of Ag NW-based films fabricated by Mayer-rod coating, spin coating, spray coating, and vacuum filtration methods are analyzed and compared. First of all, all these four methods are promising, scalable method for the fabrication of transparent electrodes, without the need for complex conditions and high cost. The obtained Ag NW-based films show high transparency and excellent mechanical flexibility. Using the same PET substrates, the Ag NW-based films fabricated by Mayer-rod coating and spray coating methods are uniform in the whole film. The deformation of the flexible substrate and the effect of centrifugal force in the spin coating process leads to the uneven distribution of Ag NWs on a large scale. Additionally, the size of obtained Ag NW-based films is restricted by the size of the membrane used in vacuum filtration process. However, this film possesses the smoothest surface after the vacuum and press procedure. As a consequence, Mayer-rod coating and spin coating methods could be used to prepare uniform films with desired size and morphology. In contrast, a precious device with a small size and thickness needs to be prepared through vacuum filtration and spin coating method. For the optoelectronic property of Ag NW-based films, these films have similar sheet resistance of 20 Ω to 250 Ω and transparency of 90% to 99%. After being prevented with PMMA layers, the Ag NW/PMMA films show excellent stability after a repeat bending test for 10,000 cycles. Moreover, the film heater could reach 72.5 °C in 50 s at a voltage of 5 V. The study of different methods for preparing Ag NW-based films in this paper can provide a reference for the selection of fabricating methods in varied application fields.

## Data Availability

All data generated and analyzed are included within this research article.

## Supplementary Materials

The following supporting information can be downloaded at: https://www.mdpi.com/article/10.3390/molecules27248907/s1, Figure S1: (a) Diameter and (b) length histograms showing the distribution of the Ag nanowires after purification; Figure S2: Detailed photos of films fabricated by (a) Mayer-rod coating method, (b) spin coating method, (c) spray coating method and (d) vacuum filtration method; Figure S3: SEM images of Ag NW-based films prepared through vacuum filtration, Mayer-rod, spin coating and spray coating methods, respectively; Figure S4: SEM images of Ag NW-based films prepared through vacuum filtration, Mayer-rod, spin coating and spray coating methods, respectively; Figure S5: Optical transmittance spectra of the Ag NWs FTCFs fabricated with different methods; Figure S6: Diagrams of the sheet resistance maps of Ag NW-based FTCFs. The orange circle represents the Ag NW-based FTCFs fabricated by vacuum filtration method; Figure S7: (a) Plots of optical transmittance (*λ* = 550 nm) versus sheet resistance for PET/Ag NW/PMMA films fabricated by four methods. (b) Plots of 1/Rs versus *T*(*λ*)^−0.5^ − 1 for the PET/Ag NW/PMMA films fabricated by four methods; Figure S8: (a) Photography of the Ag NW-based FTCFs with size of 25 cm × 20 cm fabricated by Mayer-rod coating method. (b) Photography of the Ag NW-based FTCFs with size of 36 cm × 25 cm fabricated by spin coating method.

## Sample Availability

Samples of the compounds are available from the authors.
