# Supplementary material for: Comparative Study on Preparation Methods for Transparent Conductive Films Based on Silver Nanowires
Source: Molecules. 2022 Dec 14;27(24):8907. doi: 10.3390/molecules27248907 (PMC9781453; doi:10.3390/molecules27248907)
Supplement: Supplementary file 1 [file molecules-27-08907-s001.zip › molecules-2066043-supplementary.pdf]

molecules

MDPI

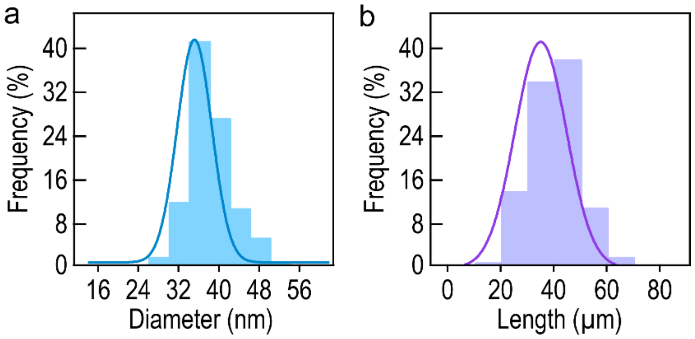

Figure S1. (a) Diameter and (b) length histograms showing the distribution of the Ag nanowires after purification.

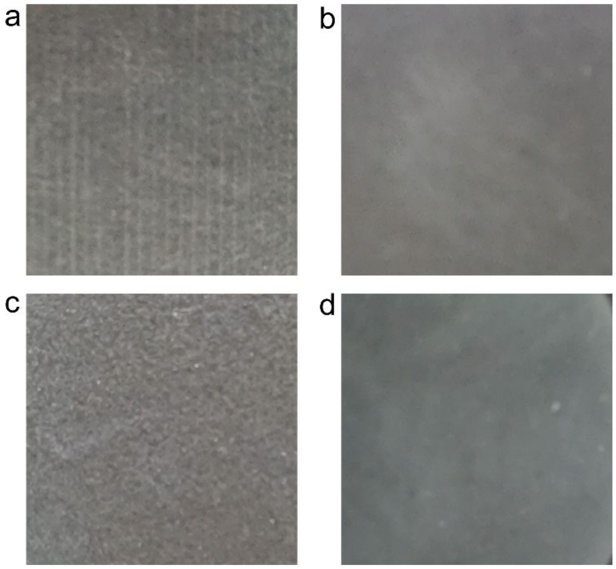

Figure S2. Detailed photos of films fabricated by (a) Mayer-rod coating method, (b) spin coating method, (c) spray coating method and (d) vacuum filtration method.

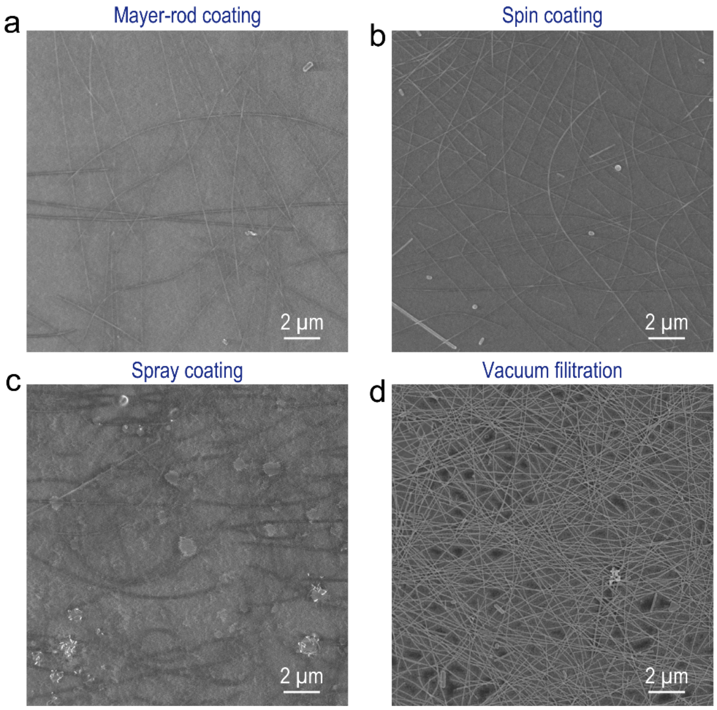

Figure S3. SEM images of Ag NW-based films prepared through vacuum filtration, Mayer-rod, spin coating and spray coating methods, respectively.

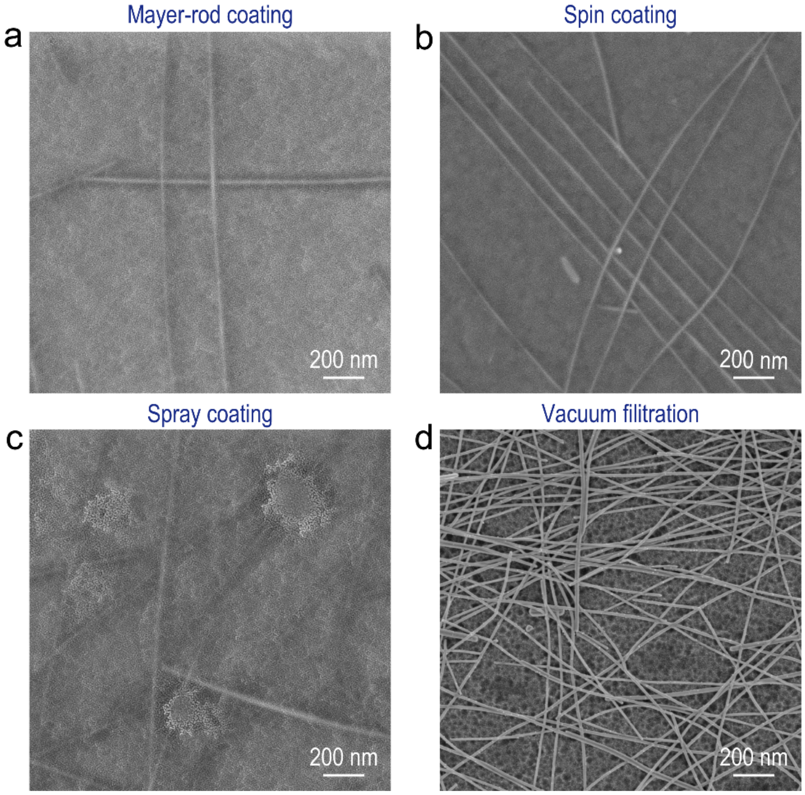

Figure S4. SEM images of Ag NW-based films prepared through vacuum filtration, Mayer-rod, spin coating and spray coating methods, respectively.

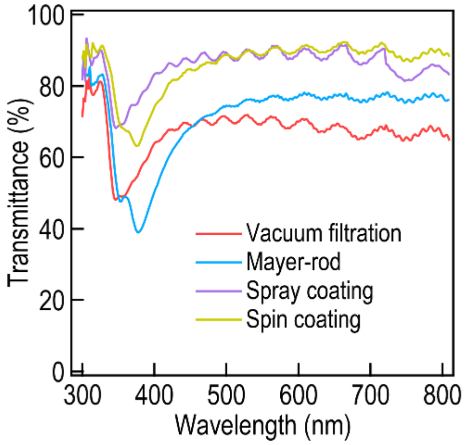

Figure S5. Optical transmittance spectra of the Ag NWs FTCFs fabricated with different methods.

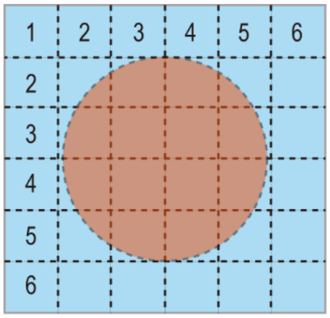

Figure S6. Diagrams of the sheet resistance maps of Ag NW-based FTCFs. The orange circle represents the Ag NW-based FTCFs fabricated by vacuum filtration method.

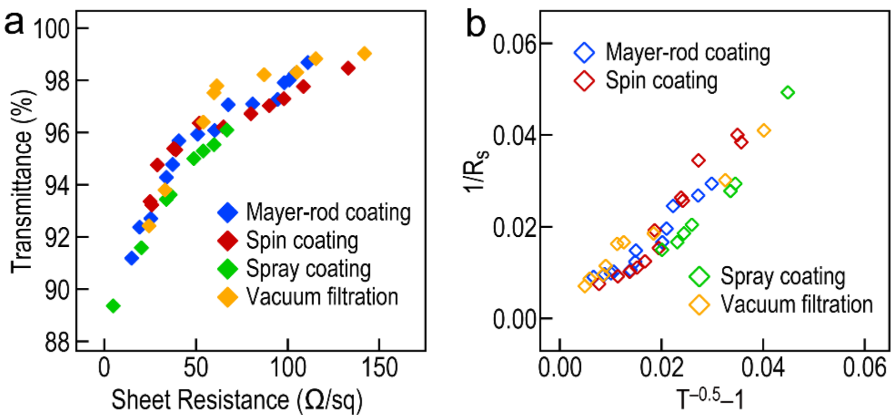

Figure S7. (a) Plots of optical transmittance (λ = 550 nm) versus sheet resistance for PET/Ag NW/PMMA films fabricated by four methods. (b) Plots of $1/R_s$ versus $T^{-0.5}–1$ for the PET/Ag NW/PMMA films fabricated by four methods.

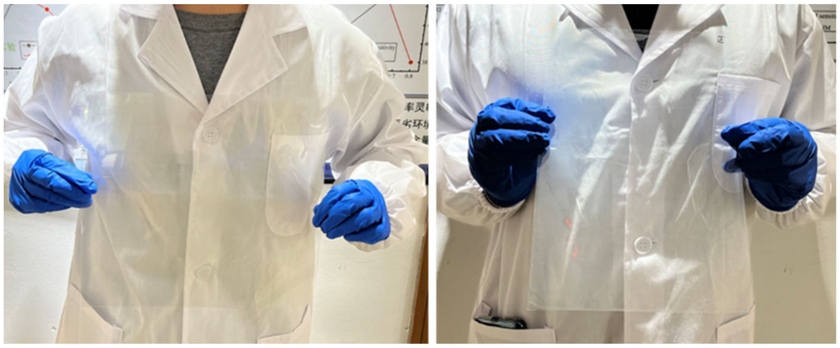

Figure S8. (a) Photography of the Ag NW-based FTCFs with size of 25 cm*20 cm fabricated by Mayer-rod coating method. (b) Photography of the Ag NW-based FTCFs with size of 36 cm*25 cm fabricated by spin coating method.
